# Chlorogenic acid mitigates acute respiratory distress syndrome via inhibition of the PI3K/AKT signalling pathway: an integrated analysis of bioinformatics and validation experiments

**DOI:** 10.1080/07853890.2025.2588840

**Published:** 2025-11-26

**Authors:** Jie Wei, Guan Ye Nai, Min Wu, Yu mei Huang, Zhao Ping Gan, Zhen Bin Wei, Hui Li, Wei Jie Zhou, Rong rong Liu

**Affiliations:** ^a^ Departments of Hematology, the First Affiliated Hospital of Guangxi Medical University, China; ^b^ Departments of hematology, Affiliated Hospital of Youjiang Medical University for Nationalities, Baise, Guangxi, China; ^c^ National Immunological Laboratory of Traditional Chinese Medicine, China; ^d^ Department of Intensive Care Unit, Baise people’s hospital, China; ^e^ Central Laboratory, Baise People’s Hospital, China

**Keywords:** Chlorogenic acid, acute respiratory distress syndrome, PI3K/AKT signaling pathway, neutrophil extracellular trap, anti-inflammation

## Abstract

**Objective:**

High mortality rates are linked to acute respiratory distress syndrome (ARDS), a prevalent type of respiratory failure. Amid the COVID-19 outbreak in particular, a viable defensive method is provided by traditional Chinese medicine (TCM). This study investigated whether chlorogenic acid (CGA), a primary component of honeysuckle, could protect against ARDS.

**Methods:**

We employed network pharmacology to explore the honeysuckle and ARDS component-target-disease network, and enrichment function analysis to uncover the potential mechanisms of honeysuckle in treating ARDS. LPS-induced ARDS rat models (each group rats *n* = 6) were used for validation, and the CGA treatments group were was administered by gavage at 100 mg/kg. including flow cytometry for T cell subsets, ELISA for inflammatory factors, and neutrophil extracellular trap (NET) markers. Histological, immunofluorescence, and transmission electron microscopy analyses were conducted to evaluate CGA’s role of CGA in ARDS. mRNA sequencing and molecular docking and surface plasmon resonance (SPR) analysis were performed to determine CGA’s influence on the PI3K/AKT signalling pathway.

**Results:**

We identified 144 common drug-disease targets, with honeysuckle containing 23 potentially active components. Key genes included STAT3, PIK3CA, and AKT1, which are involved in the PI3K/AKT, HIF-1, and Ras signalling pathways. Compared to the control group, *in vivo* studies revealed a marked diminution by CGA in cellular infiltration, oedema, and interstitial thickness observed in lungs impacted by ARDS. Furthermore, inflammatory mediators like IL-6, IL-1β, TNFα, IL-10 were lowered through CGA administration, alongside NET indicators including PAD4, citH3, and myeloperoxidase (MPO). T cell subtypes were altered during ARDS and CGA intervention. Molecular docking indicated a strong binding of CGA to PI3K and AKT1. SPR analysis further confirmed a high-affinity binding between CGA and PI3K, characterized by a low equilibrium dissociation constant (KD).

**Conclusion:**

CGA alleviates ARDS by inhibiting the PI3K/AKT signaling pathway, thereby suppressing inflammation, regulating T-cell subtypes, and reducing NET formation.

## Graphical Abstract

**Figure:**
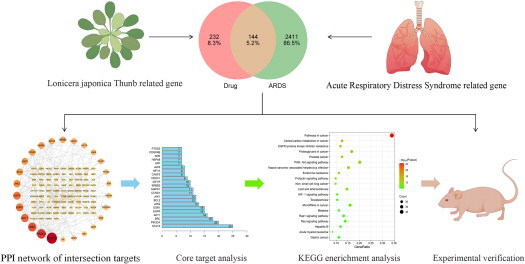

## Introduction

Elevated risks of lung-related complications arise in cancer patients, originating from side effects of medications or infiltration by malignant cells [1]. A cross-sectional analysis conducted in 2016 across 70 intensive care units revealed active cancer in 18% of ICU entries (interquartile range 9,3) [1]. Rising cancer occurrence, influenced by enhanced cancer treatment and survival rates, screening initiatives, lifestyle elements, and an aging populace, is expected to elevate these numbers [2]. Among immunocompromised individuals, severe infections affecting the respiratory tract primarily lead to intensive care unit (ICU) placements; such persons are susceptible to sepsis alongside hypoxemic acute respiratory failure (ARF) [3]. ARDS stands out as a highly severe disorder linked to substantial death rates, even under ICU management [4]. Marked by advancing breathlessness, resistant low oxygen levels, and swelling in lung tissues, ARDS represents an acute medical crisis. Triggers commonly involve gastric content aspiration, pneumonia, severe injuries, or sepsis. In the United States, ARDS impacts about 200,000 people yearly, causing close to 75,000 fatalities and surpassing worldwide mortality from HIV infection or breast cancer [5]. Globally, it influences roughly 3 million individuals each year, representing 10% of ICU intakes and 24% of those under therapy [6]. ARDS incidence is considerably greater in hematologic malignancies relative to other conditions, attributable to distinctive immune traits and application of targeted therapies, cellular immunotherapy, and radiotherapy. Pathophysiologically, ARDS involves extensive lung damage provoked by extrapulmonary and intrapulmonary elements over a limited duration, displaying histologic indicators of widespread alveolar harm that encompass alveolar bleeding, inflammation, hyaline membrane development, and pulmonary swelling [7]. Therapeutic approaches for ARDS stay constrained despite extensive studies over decades, mainly involving control over the underlying illness and Extracorporeal life support (ECLS), with persistent high fatality rates between 53.9-54.4% [8]. In addition to the control of the primary pathologies that lead to ARDS such as bacterial, fungal, and viral infections are important in the treatment of ARDS, inhibiting lung injury from the lung pathomechanisms is a great challenge in the treatment of ARDS is also intervene in the development and progression of ARDS from the aetiology of the disease. However, existing therapeutic strategies fail to exhibit definitive efficacy, particularly in individuals afflicted with hematologic malignancies. Immunosuppressive procedures commonly induce agranulocytosis during treatment, arising from recurrent septic events, pulmonary infections, and additional factors capable of precipitating ARDS. As a result, discovering innovative interventions emerges as a pressing priority to mitigate pulmonary damage, thereby enabling management of disease advancement in ARDS-affected persons while lowering fatality rates.

ARDS plays a significant regulatory role in immune cells during pathological processes such as alveolar injury and inflammatory exudation in lung lesions. Immediate activation is triggered within the innate immune system upon pathogen entry into the host [9]. Epithelial cells of the lungs, together with those of the innate immune system, exhibit expression of pattern recognition receptors (PRRs) [10]. Detection of intruding pathogens by PRRs occurs through damage-associated molecular patterns (DAMPs) alongside pathogen-associated molecular patterns (PAMPs). A cytokine storm emerges as the immune reaction undergoes amplification and initiation throughout this sequence. Dysfunction in vascular endothelium stems from extensive discharge and production of pro-inflammatory agents induced by the cytokine storm; this process subsequently heightens permeability within pulmonary microvessels, thereby promoting both onset and advancement of ARDS [11]. Accordingly, stimulation across diverse pathways for inflammatory signaling, including NF-κB and JAK/STAT, becomes evident among individuals suffering from ARDS precipitated by sepsis and virus[12–14]. Injury affecting alveolar endothelial cells and those of the mesenchyme is provoked by inflammation amid the initial pathologic phase of ARDS. Research by Englert et al. demonstrated that cytokine storms arising from sepsis might induce indirect impairment to endothelium in the lungs or direct impairment to epithelium in the lungs [15^,^]. Because of the different causes of ARDS, its treatment needs to be individualized according to different patients. Several studies on ARDS treatment have focused on the prognostic impact of mechanical ventilation. Drugs commonly used in the treatment of ARDS include neutrophil elastase inhibitors to inhibit neutrophil-induced lung injury, the efficacy of which has been demonstrated in COVID-19-induced ARDS [4,16], and ketoconazole to inhibit inflammation, thereby alleviating the progression of ARDS [17]. Thus, uncontrolled inflammation is a central problem in ARDS, and understanding how inflammation is modulated is critical for developing new therapeutic strategies to limit excessive inflammation.

Various herbal preparations have been introduced to treat ARDS induced by novel coronavirus infection at the stage of novel coronavirus pneumonia, and their therapeutic effects have been encouraging [18,19]. Honeysuckle, utilized within traditional Chinese medicine, demonstrates abilities for evacuating wind-heat alongside clearing heat to detoxify toxins. Anti-inflammatory, antiviral, and antioxidant properties have been revealed for honeysuckle through pharmacological investigations [20–22]. The study’s results by Liu C et al. demonstrated that honeysuckle could treat a mouse model of phospholipopolysaccharide-induced ARDS, but the specific molecular mechanism has not yet been clarified. Because honeysuckle and its active ingredients can treat diseases, they can not only treat ARDS but also play a protective role in lung damage to target organs caused by the cause[21]. In this investigation, potential safeguarding effects against lung damage in ARDS triggered by LPS were examined for CGA, serving as the principal effective constituent extracted from honeysuckle, by employing techniques including experimental validation, molecular docking, and network pharmacology.

## Materials and methods

### 1. Experimental materials

Chlorogenic acid (CA; Cat. C3878-1G; Sigma-Aldrich), LPS (Cat No. ST1470, Biotronix), and TNF-α (cat. D731168; Bioengineering) and IL-1β (Cat No. D731007; Bioengineering) and IL-6 (Cat No. D731010, Bioengineering), IL- 10 (Cat No. D731011, Bioengineering), and CitH3 (Cat No. ELK0876, Kolu), and PAD4 (Cat No. SP13578, Saipex). p-PI3K (Cat No. A22996, ABclonal), and p-PI3K (cat. AP1463; ABclonal), AKT (cat. A18675; Abcam), and p-AKT (Cat No. AP1430, ABclonal), β-actin (Cat No. 60008-1-Ig, Proteintech), CD4 (Cat No. FITC-65104, Proteintech), CD8 (Cat No. 65205-1-Ig, Proteintech), CD25 (Cat No. 17-0251-82, Thermo Fisher Scientific), Foxp3 (Cat No. PE-65089, Proteintech) and MPO (Cat No. 81610-1-RR. Proteintech), Citrullinated Histone H3 (Cat No. 13754-1-AP, Proteintech), HRP goat anti-rabbit IgG (Cat No. AS014, Abclonal), HRP goat anti-rabbit IgG (Cat No. AS003, Abclonal), TRIzol (Cat No. 15596018, Thermo Fisher), RNA extraction kit (Cat No. 12183025, Thermo Fisher), Oligo-dT (Cat No. 18418012, Thermo Fisher), dNTPs (Cat No. 18427088, Thermo Fisher), ReverTra Ace qPCR RT Master Mix (Cat No. FSQ-301, TOYOBO), and SYBR Premix (Cat No. 4368708, Thermo Fisher Scientific).

### 2. Network pharmacology analysis

#### 2.1. Drug composition and target screening

Systematic collection of effective elements from honeysuckle (Lonicera japonica Thunb.) was conducted through the Traditional Chinese Medicine Systems Pharmacology Database and Analysis Platform (TCMSP, https://old.tcmsp-e.com/). Presumed objectives for those substances were then obtained *via* the TCMSP database before undergoing confirmation with the SwissTargetPrediction server (http://www.swisstargetprediction.ch/), whereby forecasts possessing probability values greater than 0 were exclusively preserved.

#### 2.2. Disease target screening

Genes linked to ARDS were assembled by employing ‘acute respiratory distress syndrome’ as the keyword across a set of three extensive repositories, namely Disgenet (https://www.disgenet.org/), Genecards (https://www.genecards.org/), and Online Mendelian Inheritance in Man (OMIM, https://omim.org/).

#### 2.3. Screening of drug-disease common targets

Potential therapeutic objectives emerged from common intersections amid constituents derived from honeysuckle and genes associated with ARDS; depiction of these was accomplished through employment of an interactive tool for Venn diagrams (https://bioinfogp.cnb.csic.es/tools/venny/).7

#### 2.4. Construction and analysis of traditional Chinese medicine-component-target-disease network

Employment of Cytoscape software (version 3.9.1) enabled both creation and illustration of a network involving disease, targets, and compounds. Assessment through degree centrality, derived *via* the incorporated NetworkAnalyzer feature, allowed for recognition of nodes exhibiting topological prominence within this network, encompassing core targets alongside active compounds.

#### 2.5. PPI network construction and core target analysis

To elucidate the interactions among the common targets, a Protein-Protein Interaction (PPI) network was generated using the STRING database (version 11.5, https://string-db.org). The search was confined to Homo sapiens, with a minimum required interaction score set to 0.900 (highest confidence). Isolated nodes not connected to the main network were excluded from the final visualization.

#### 2.6. GO and KEGG enrichment analysis

Employment of the Database for Annotation, Visualization, and Integrated Discovery (DAVID, version 6.8) enabled execution of both pathway enrichment examination and functional labelling aimed at shared objectives. Examination covered pathways from the Kyoto Encyclopedia of Genes and Genomes (KEGG) together with categories in Gene Ontology (GO) that incorporated molecular functions (MF), cellular components (CC), and biological processes (BP). Entries displaying a false discovery rate (FDR) under 0.05 received designation as possessing statistical importance. Depiction of findings took shape through bubble charts alongside bar diagrams by leveraging the ggplot2 module embedded in R (version 4.2.1).

#### 2.7. Molecular docking

Acquisition of the three-dimensional configuration for Chlorogenic Acid (CID: 1794427) in SDF format occurred from the PubChem database (https://pubchem.ncbi.nlm.nih.gov/), with subsequent energy minimization achieved through application of MMFF94 force fields within Open Babel. From the RCSB Protein Data Bank (www.rcsb.org), crystalline architectures of AKT1 (PDB ID: 6HHF) alongside PI3K (PDB ID: 6O9G) were sourced. Execution of protein readiness, which involved charge assignment, hydrogen incorporation, and water elimination, took place *via* AutoDockTools (ADT) 1.5.6. Simulations pertaining to molecular docking were implemented by means of AutoDock Vina 1.1.2. Definition of the binding area entailed a grid enclosure that surrounded the recognized active region within every protein. Selection for additional evaluation was made of the structure displaying the most advantageous (minimal) binding affinity.

#### 2.8. Molecular dynamics (MD) simulations of the protein–ligand complexes

Execution of molecular dynamics (MD) simulations targeting complexes between ligands and proteins transpired across a 100 ns period through the utilization of GROMACS 2020.6. Topology creation for the protein was accomplished *via* the AMBER99SB-ILDN force field, whereas ligand topology development occurred with assistance from the Generalized Amber Force Field (GAFF) integrated into Amber20. Immersion of the system took place inside a TIP3P water container configured as a truncated octahedron that included a 10 Å margin, coupled with charge neutralization achieved by incorporating Cl^-^/Na^+^ counterions. The procedure for energy minimization unfolded across dual consecutive segments: commencing with 2,500 iterations of steepest descent, then advancing to 2,500 steps involving conjugate gradient refinement. Thereafter, system stabilization ensued within isothermal-isobaric (NPT) alongside canonical (NVT) ensembles over durations of 100 ps apiece, during which the V-rescale thermostat governed temperature at 300 K. Throughout NPT stabilization, the Parrinello-Rahman barostat preserved pressure levels at 1 bar (101.325 kPa). Primary MD computations extended to 100 ns amid periodic boundary settings, featuring trajectory captures at intervals of 10 ps. Subsequent to simulation, computations of root-mean-square fluctuation (RMSF) in conjunction with root-mean-square deviation (RMSD) were obtained from trajectory datasets to evaluate residue adaptability and structural constancy of the system.

#### 2.9. Surface plasmon resonance (SPR) analysis

Evaluation of the affinity for binding amid Chlorogenic acid and PI3K occurred *via* surface plasmon resonance applied to a CM5 sensor chip. Immobilization of the ligand (PI3K) took place upon the sensor chip, whereas injection of the analyte (Chlorogenic acid) proceeded at diverse concentrations for the purpose of determining parameters related to kinetics.

##### Ligand immobilization

Dilution of PI3K to a concentration reaching 20 μg/mL took place within an immobilization buffer composed of 10 mM sodium acetate at pH 4.5, after which fixation onto the sample channel (Fc2) ensued by means of amine coupling. A blend incorporating 0.1 M NHS together with 0.4 M EDC facilitated activation of the chip across a span of 420 s, under a flow velocity set at 10 μL/min. Attainment of the immobilization threshold approximated 12,600 response units (RU). Absence of alterations characterized the reference channel (Fc1). Utilization of 1 M ethanolamine hydrochloride enabled deactivation of remaining reactive sites.

##### Analyte binding kinetics

Serial dilution of Chlorogenic acid took place inside an analyte buffer that included pH 7.4, 0.005% Tween 20, 1% DMSO, and 1× PBS, yielding eight separate levels that extended between 0.01 and 0.50 μM. Delivery of individual levels occurred upon Fc2 channels in conjunction with Fc1 at a velocity reaching 20 μL/min for the flow. Tracking of the association period lasted across 100 s, with the dissociation period subsequently commencing over 180 s. Amid consecutive sequences, renewal of the chip ensued through application of 10 mM glycine-HCl (pH 2.0) administered at 200 μL/min throughout 30 s. Completion of each trial was executed under conditions set at 22 °C.

##### Data analysis

Utilization of the SPR evaluation software enabled sensorgram processing alongside alignment to a 1:1 interaction paradigm. Determination was achieved for indicators of kinetics that incorporated dissociation rate constant (kd), equilibrium dissociation constant (KD), and association rate constant (ka).

### 3. Validation experiments

#### 3.1. Rat ARDS model construction

Procurement of male Sprague Dawley (SD) rats, possessing body weights that spanned roughly from 240 to 260 g, occurred through acquisition from Shanghai Slaughter Laboratory Animal Co. Allocation at random distributed these rodents across categories labelled as control, ARDS+CGA intervention (entailing 100 mg/kg delivered by means of sustained intragastric administration spanning 7 days), and ARDS, featuring 6 specimens within every division; formulation entailed solubilization within sterilized saline to yield a concentration reaching 1 mg/mL. Induction of the ARDS paradigm was accomplished *via* delivery of an isolated intraperitoneal (i.p.) administration involving Lipopolysaccharide (LPS obtained from Escherichia coli O55:B5) dosed at 2 mg/kg. This dose was selected based on pilot studies confirming robust lung inflammation and injury at 24 h post-injection. To evaluate the therapeutic potential of CGA, treatment was initiated 4 h after LPS challenge, mimicking a post-onset intervention strategy. Chlorogenic acid (100 mg/kg, dissolved in sterile saline) or vehicle was administered daily *via* intragastric gavage for a total of 7 days. Serum and lung tissues were collected for subsequent experiments. At the end of the experiment, euthanasia was performed using the cervical dislocation after adequate anaesthesia. This method complies with the recommended standards for rodents in the ‘Animal Euthanasia Guidelines’ of the American Veterinary Medical Association (AVMA) and has been approved by the Laboratory Animal Ethics Committee of Youjiang Medical University for Nationalities.

#### 3.2. mRNA sequencing

Determination of RNA purity alongside concentration ensued subsequent to extraction from a segment of pulmonary tissue. Enrichment for mRNA possessing a polyA tail was accomplished through application of Oligo(dT) magnetic beads, with the acquired mRNA undergoing random fragmentation induced by divalent cations within the NEB Fragmentation Buffer. Generation of the initial cDNA strand occurred inside the M-MuLV reverse transcriptase system by utilizing fragmented mRNA to serve as the template while employing random oligonucleotides to act as primers. Breakdown of the RNA filament was executed *via* RNase H, after which construction of the secondary cDNA filament proceeded with dNTPs integrated into a DNA polymerase I setup. Terminal restoration was implemented on the purified double-stranded cDNA. Sequencing adapter ligation, A-tail incorporation, and terminal restoration preceded filtration of cDNA fragments that spanned 250–300 bp by means of AMPure XP beads, followed by augmentation through PCR and renewed cleansing of the amplification results utilizing AMPure XP beads to secure the ultimate collection. Sequencing directed at the transcriptome was carried out on an Illumina HiSeq system after assembly of the library had concluded.

#### 3.2. Flow assay of T cell subsets

Single-cell suspension was achieved through filtration subsequent to pulverization of a segment isolated from murine pulmonary tissue. Application of a lysing agent targeted at erythrocytes facilitated their elimination, followed by execution of density modifications alongside cellular enumeration. The flow antibody mixture was prepared with FACS buffer according to the dilution ratio of the antibody instruction, and the staining was carried out by avoiding light, and analyzed by BD Fortessa after the staining was finished, and the data processing software was FlowJo Version 10.0.

#### 3.3. ELISA for common inflammatory factors and neutrophil trapping network markers of inflammation

Adherence to guidelines provided within the ELISA kit occurred for evaluations encompassing PAD4, CitH3, IL-10, IL-6, IL-1β, and TNF-α. Deployment of the standard substance facilitated creation of a concentration gradient, alongside construction of the standard curve derived from recorded OD readings, which permitted ascertainment of relevant concentrations through alignment with sample absorbance values on the graphical axis.

#### 3.4. HE staining

Pulmonary tissues underwent embedding subsequent to fixation, followed by sectioning, deparaffinization, haematoxylin staining, differentiation *via* ethanol hydrochloride, eosin staining, achievement of transparency through dehydration, sealing, and drying. Microscopic assessment ensued thereafter to evaluate modifications in pathologic architecture present within the lung tissues.

#### 3.5. Immunofluorescence

Tissue sections were baked, deparaffinized, permeabilized, antigenically repaired, closed, diluted according to the recommended dilution of the antibody specification, incubated with primary antibody and corresponding immunofluorescence secondary antibody and fluorescence quencher for sealing, photographed, and observed under a fluorescence microscope.

#### 3.6. Transmission electron microscopy

Lung tissue was fixed with 2% glutaraldehyde, washed with MPBS buffer for dehydration and replacement, and dried at the CO2 critical point. After drying, the samples were subjected to a normal gold spray coating operation to enhance their visibility and resolution under an electron microscope. Finally, the processed lung tissue samples were examined under an electron microscope for scanning and observation.

#### 3.7. Western blot assay

An appropriate amount of lung tissue was collected, and after adding RIPA protein lysate for sufficient grinding, the supernatant, that is, the total tissue protein, was obtained by separation in an ultra-high-speed centrifuge pre-cooled at 4 °C. Quantification of the protein that had been isolated occurred through application of the BCA kit designed for protein measurement. Thereafter, dilution of secondary alongside primary antibodies proceeded in alignment with directives supplied by the producer, while SDS-PAGE electrophoresis was conducted in conjunction with membrane translocation, ultimately leading to visualization *via* ECL.

##### Ethical statements

Approval for the present investigation was obtained from the Institutional Review Board affiliated with Youjiang Medical University for Nationalities (No. 2024-YY-854). Every protocol associated with animals was implemented in alignment with tenets from the Guide for the Care and Use of Laboratory Animals in conjunction with those from the Declaration of Helsinki. Compliance with the ARRIVE directives was ensured in this research.

## Results

### Drug, disease target, and drug-disease co-target screening

**Figure 1.**
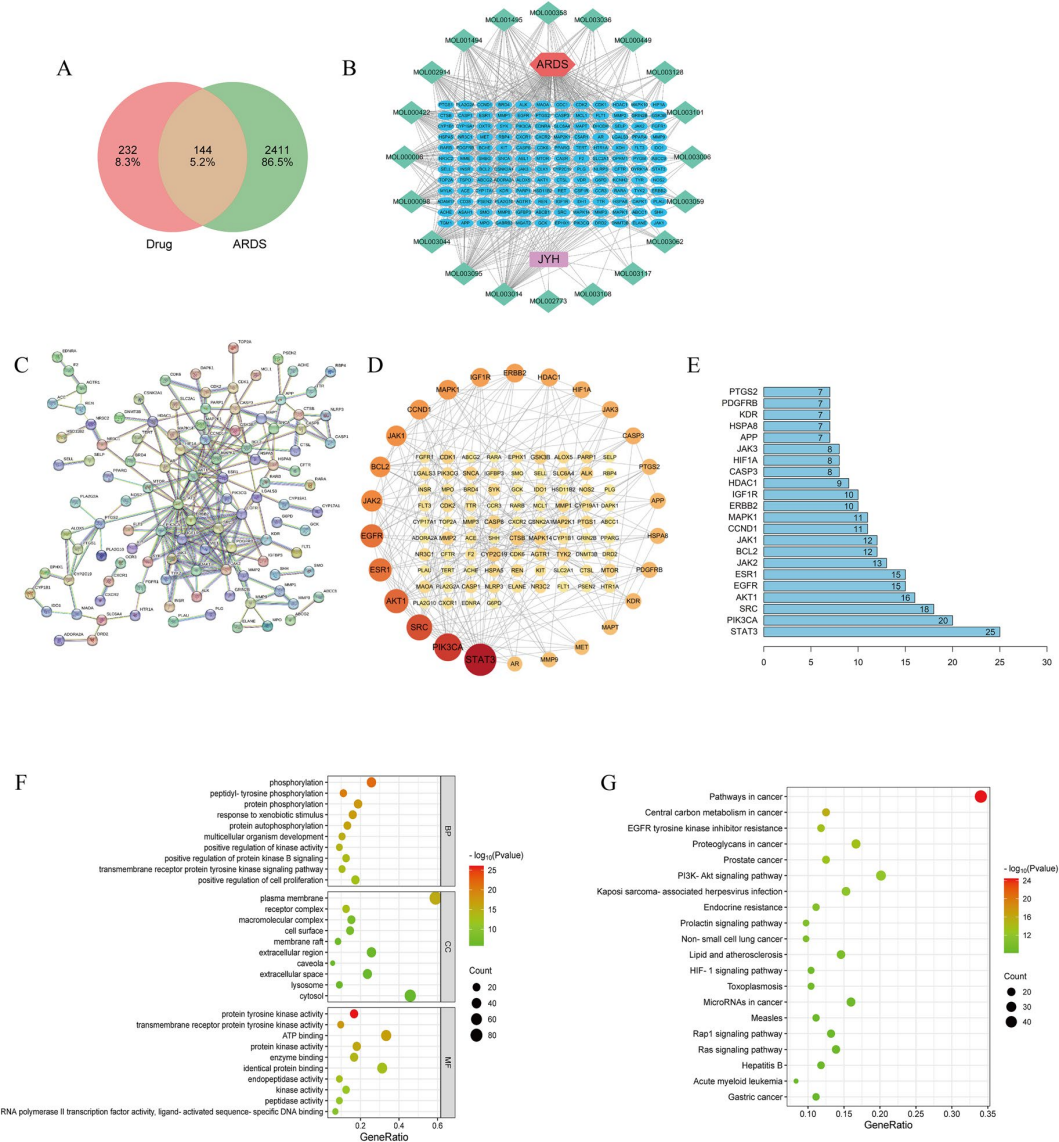
The network pharmacology between ARDS and JYH. A: The common targets of ARDS and JYH; B the JYH componts and the ARDS tagerts; C-E the PPI network of common targets and the hub genes; F-G: Enrichment analysis of hub genes.

Identification of effective elements derived from honeysuckle transpired through utilization of the TCMSP database, incorporating thresholds like DL ≥ 0.18 in conjunction with OB ≥ 30%, which culminated in the acquisition of 23 prospective active substances overall. Determination of objectives was facilitated by engagement with the Swiss Target Prediction repository, yielding 376 pharmaceutical objectives subsequent to elimination of redundancies. By searching the OMIM, Disgenet, and Genecards databases with the keyword “acute respiratory distress syndrome,” we obtained 2,555 disease targets, which were also de-duplicated. Using the Venny2.1 online software, a Venn diagram was constructed by inputting 376 drug targets and 2,555 disease targets, revealing 144 drug-disease common targets (Figure 1(A)).

### Construction and analysis of the Chinese medicine-component-target-disease network

Integration of the 144 shared targets between diseases and drugs alongside the 23 prospective effective elements from Honeysuckle occurred within the Cytoscape application. Elimination ensued for standalone substances lacking overlap with the objectives, thereby facilitating generation of a diagram depicting the ‘TCM-constituent-target-disease’ network (Figure 1(B)). Within such a framework, diseases are denoted by red nodes, 144 mutual objectives by blue nodes, 20 functional constituents present in Honeysuckle by green nodes, and pharmaceuticals by purple nodes.

### PPI network construction and core target analysis

Shared targets amid diseases and drugs underwent integration into the STRING database to enable evaluation, incorporating designation of protein category as “Homo sapiens” in conjunction with establishment of the least interaction cutoff at 0.9. Standalone nodes subsequently experienced exclusion, which permitted development of the PPI structure aimed at protein engagements (Figure 1(C)).

The 144 common targets were also imported into the STRING database with the protein species set as ‘Homo sapiens’ and the minimum interaction threshold at 0.9. The network relationship data of target interactions were obtained and imported into Cytoscape software to draw the protein interaction network diagram (Figure 1(D)). Node size and colour shades vary according to the node’s degree value, with the outermost circle representing targets with a degree greater than five. The network comprised 111 nodes and 229 edges. The PPI network was analysed using the NetworkAnalyzer tool in Cytoscape 3.9.1, and core targets were selected based on the degree value, with higher values indicating greater importance. The top 20 targets by degree ranking were plotted using R (version 4.2.1) (Figure 1(E)).

### GO and KEGG enrichment analysis

Application of GO enrichment examination to the 144 mutual objectives transpired *via* the David repository, resulting in classification into molecular functions, cellular components, and biological processes. Findings from GO demonstrated enrichment of overlapping genes across 140 processes linked to molecular functions, 83 processes concerning cellular component expression, and 647 pathways associated with biological processes. Selection occurred for the leading 10 routes based on P values within every division to facilitate depiction through bar charts. Furthermore, implementation of KEGG enrichment scrutiny targeting the 144 shared objectives within the David repository uncovered an aggregate of 149 KEGG routes, from which the foremost 20 in terms of P-value hierarchy were chosen for illustration *via* bubble diagrams alongside bar graphs enriched for KEGG (Figure 1(F–G)).

### Flow assay of T cell subsets

**Figure 2.**
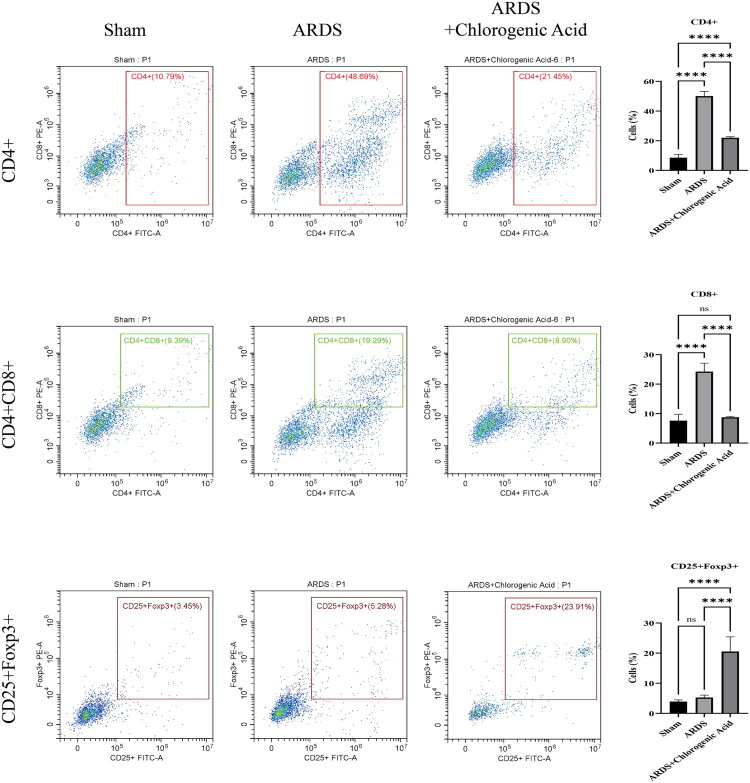
The analysis of T cells subtypes changes in ARDS and after CGA intervention.*<0.05; **<0.01; ***<0.001; ****<0.0001.

Compared to the control group, a marked elevation was observed in CD8+ and CD4+ T cells among rats modeled with ARDS induced by LPS (*p* < 0.0001). The quantity of CD25 + Foxp3+ T cells exhibited no notable variation across these two cohorts. Nevertheless, administration of CGA derived from Honeysuckle extract led to substantially reduced levels of CD8+ and CD4+ T cells relative to the ARDS cohort (*p* < 0.0001), while elevations in CD25 + Foxp3+ T cells became evident when contrasted with the ARDS model group (*p* < 0.0001) alongside the control (*p* < 0.001) (Figure 2). Such observations imply a pivotal involvement of T cells in pulmonary damage associated with ARDS in the modeled scenario, whereas honeysuckle extract CGA administration augments negatively modulated T cell functionality to inhibit CD8+/CD4+ T cells.

### Lung HE, alveolar lavage fluid, and serum ELISA assays

**Figure 3.**
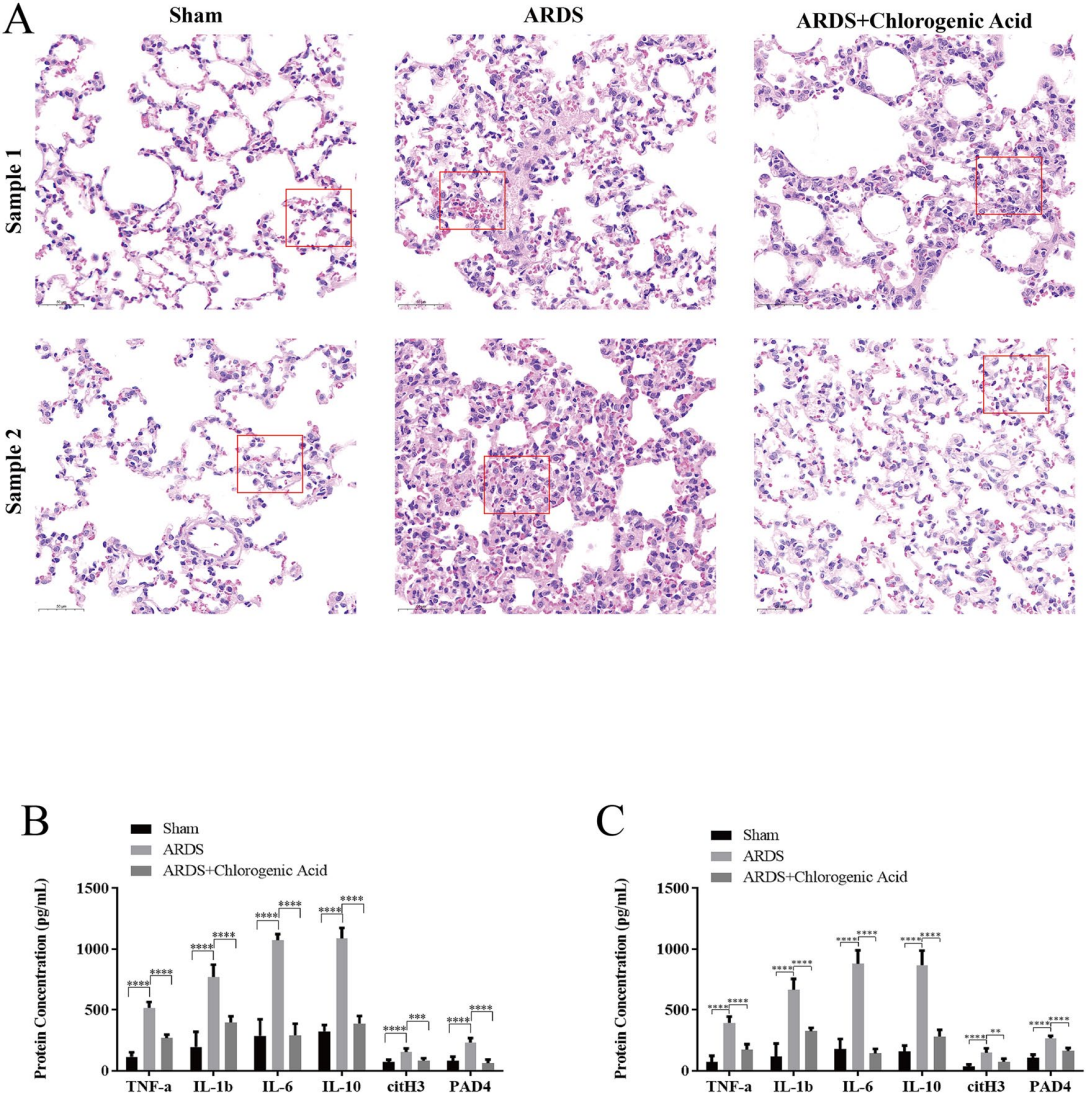
The histology and inflammation analysis. A: the histological of blank, ARDS, and ARDS+CGA groups; B: the inflammatory factors and NET markers in serum *via* ELISA; C: the inflammatory factors and NET markers in alveolar lavage fluid *via* ELISA. *<0.05; **<0.01; ***<0.001; ****<0.0001.

Investigations encompassing pathologic analysis through HE staining of lungs, alongside evaluations of NET indicators and inflammatory mediators within bronchoalveolar lavage fluid, were executed to ascertain protective influences exerted by CGA upon pulmonary damage induced by ARDS. Observations unveiled augmented extents in ARDS-impacted lungs as opposed to controls with respect to edema intensity, cellular infiltration prevalence, and interstitial thickness. On the contrary, notable declines emerged in cellular infiltration quantities, oedema magnitude, and interstitial thickness subsequent to CGA administration relative to the ARDS cohort (Figure 3(A)). Assessments *via* ELISA targeting mediators of inflammation in bronchoalveolar lavage fluid demonstrated substantially heightened concentrations for IL-10, IL-6, IL-1β, and TNFα within the ARDS category compared against controls (*p* < 0.0001). After CGA intervention, these factors were significantly decreased compared with those in the ARDS group (*p* < 0.0001). We also investigated the expression levels of the NET markers citH3 and PAD4 in the three groups and found that they were significantly higher in the ARDS group than in the control group (*p* < 0.0001). Subsequent to CGA administration, substantial reductions emerged in the levels of these indicators relative to the ARDS cohort, particularly evident for PAD4 alongside citH3 (*p* < 0.01) and MPO (*p* < 0.0001), as depicted in Figure 3(B–C). Comparable patterns materialized during evaluations of serum specimens. Observations of this nature suggest that honeysuckle’s principal effective constituent, CGA, exhibits ability to curtail formation of neutrophil extracellular traps in conjunction with inflammatory mediators within the lungs, thus affording defense against pulmonary harm induced by ARDS.

### Further determination of NET markers by immunofluorescence

**Figure 4.**
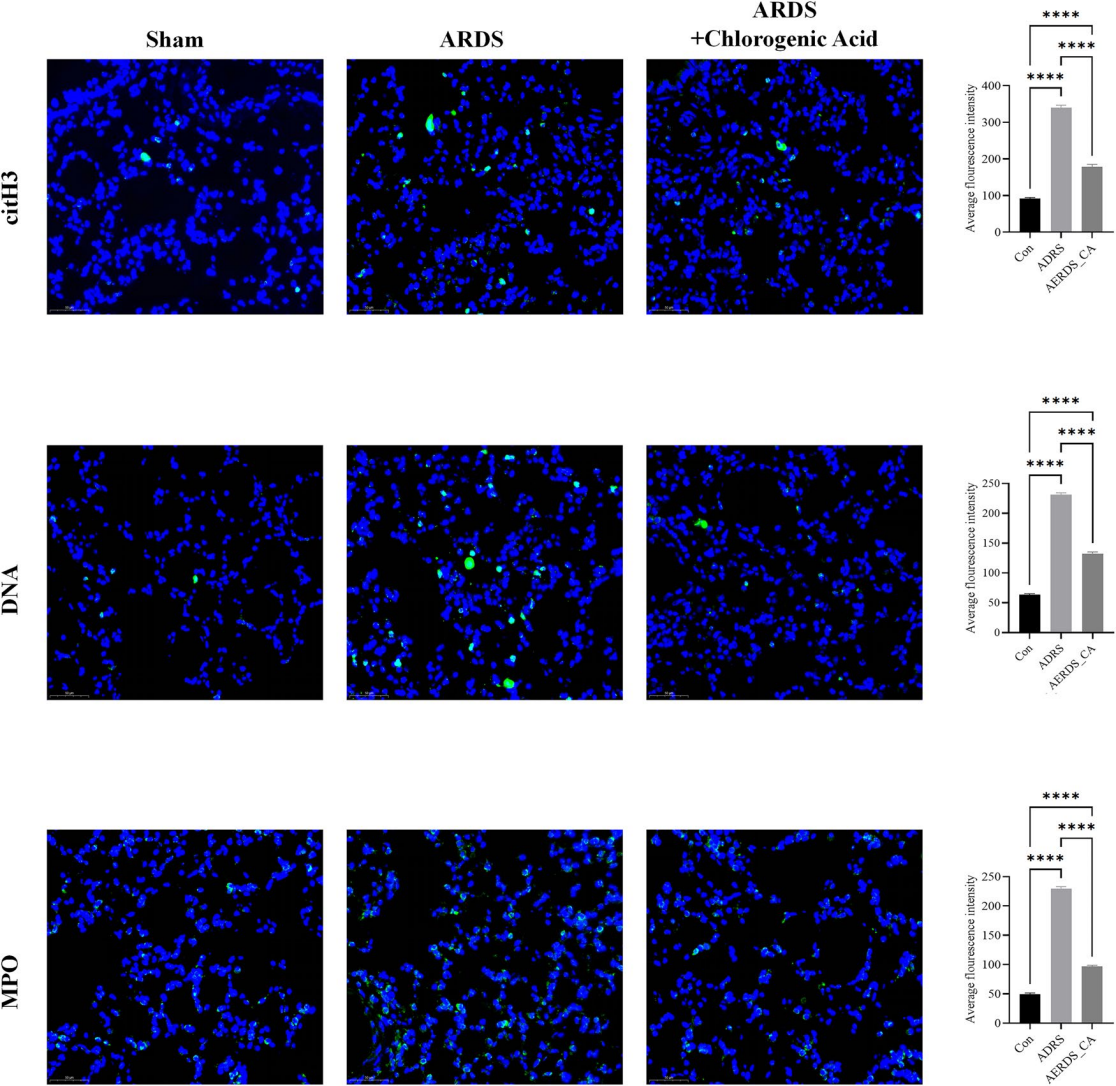
Immunofluorescence analysis of NET markers. *<0.05; **<0.01; ***<0.001; ****<0.0001.

The relationship between NET formation and ARDS lung injury has garnered increasing interest, particularly since the COVID-19 pandemic. Inhibitors of NET formation have been shown to treat COVID-19-induced ARDS lung injuries. We explored whether the main active ingredient of Honeysuckle, CGA, could inhibit NET formation and thus alleviate lung injury. To visualize NET markers at the lung tissue level more intuitively, we used fluorescent labelling for further analysis. The results showed that the ARDS group had higher levels than the control group (*p* < 0.0001), and the ARDS + CGA group had lower levels than the ARDS group (*p* < 0.0001) (Figure 4). This is consistent with the ELISA results, suggesting that CGA, the main active ingredient of Honeysuckle, exerts a protective effect against ARDS lung injury through multiple pathways.

### Transmission electron microscopy detection of subcellular structural changes in the lungs

**Figure 5.**
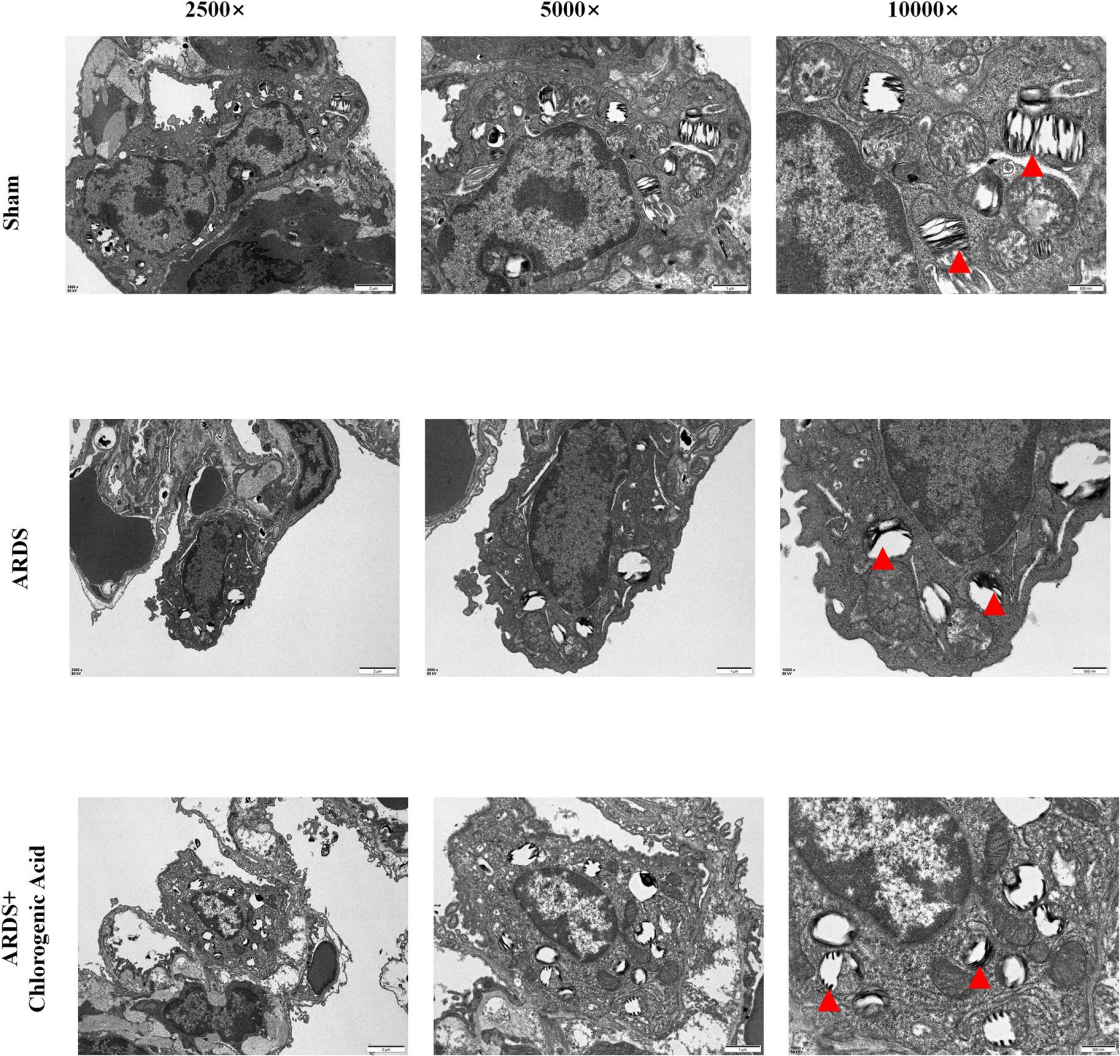
Analysis of the subcellular structure of lung tissue by projection electron microscopy.

To further understand the damage caused by ARDS to the subcellular structure of the lungs and the protective effect of the main active ingredient of Honeysuckle against ARDS, we performed transmission electron microscopy on the lung tissues of the control, ARDS, and ARDS+CGA groups. The results showed that the subcellular structure of the lung tissues in the ARDS group was disorganized, with the disappearance of the normal mitochondrial structure (Figure 5). In contrast, the ARDS + CGA group showed a normal mitochondrial structure. These results further indicate that the main active ingredients of honeysuckle have a protective effect against ARDS lung injury.

### ARDS lung injury and the possible molecular mechanism of honeysuckle’s main active ingredient CGA on lung protection

**Figure 6.**
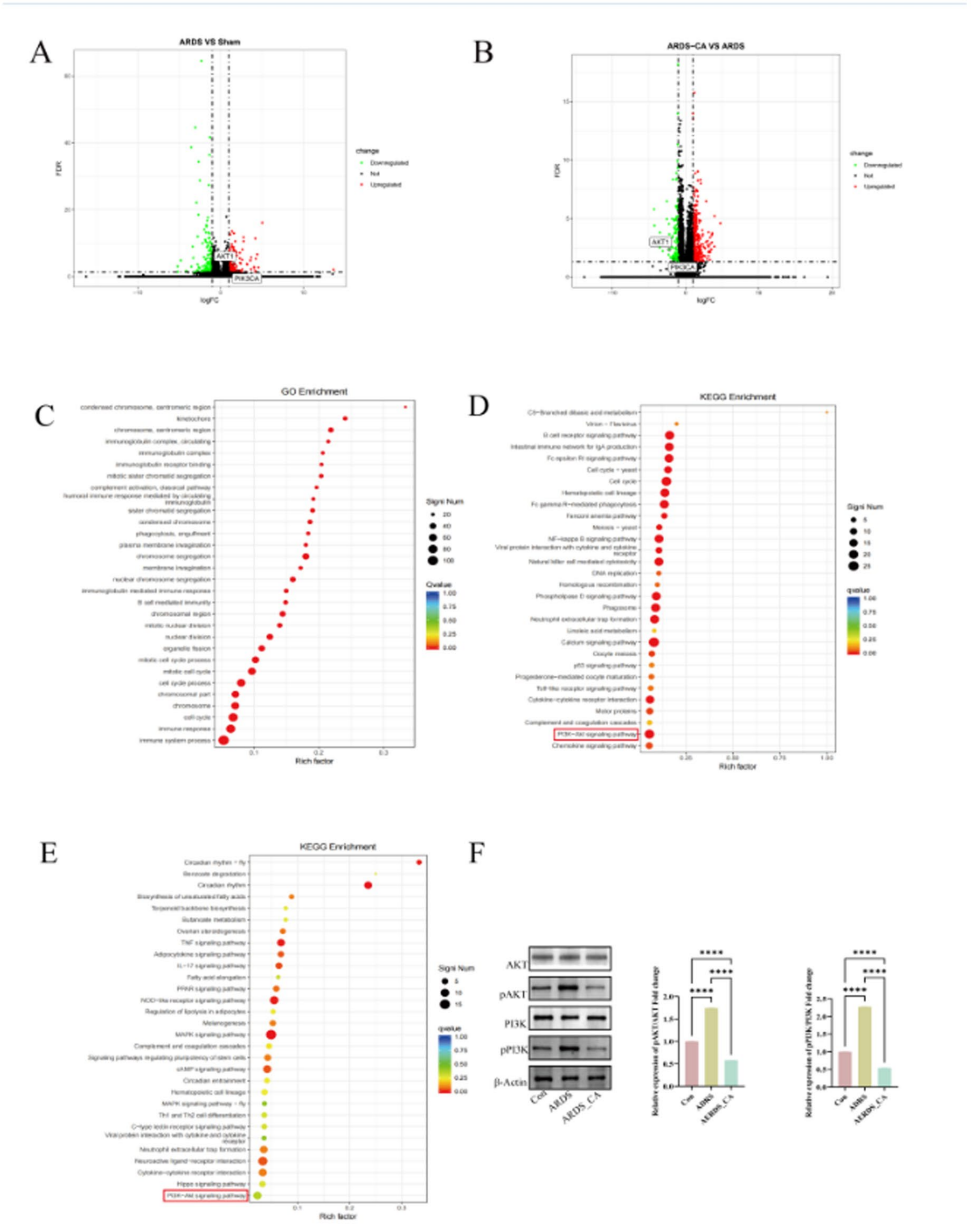
The functionality of mRNA high-throughput sequencing and molecular signaling pathway enrichment analysis. A:Principal Component Analysis; B: Volcano plot analysis of ARDS+CGA and ARDS groups; C: Go analysis of the differential genes between ARDS+CGA and ARDS groups; D: KEGG analysis of differential genes between ARDS+CGA and ARDS; E: KEGG analysis of differential genes between ARDS and sham; F: The western blot analysis of underlying pathway for ARDS progression and CGA protecting ARDS. *<0.05; **<0.01; ***<0.001; ****<0.0001.

Compared with the sham group, the ARDS group significant differential genes expression showed in the Figure 6(A). Compared to the ARDS group, the ARDS with CGA intervention with 341 and 113 up-regulated and down-regulated genes, respectively (Figure 6(B)), and the PI3K and AKT are significant expression genes. And further GO analysis of these differential expression genes showed that they as the parts of immune response, B cells mediated immune response, immunoglobolin-mediated immune response, and so on (Figure 6(C)), these results indicated these genes may be the key regulators for immune system for ARDS. In the preliminary analysis of the PPI core genes through network pharmacology, the PI3K/AKT molecular signalling pathway, including PI3KCA and AKT1, was ranked at the top. KEGG molecular signalling pathway analysis also revealed that the PI3K/AKT pathway was highly ranked. Experimental verification conducted following enrichment assessment of functions and sequencing at high throughput revealed enrichment within the PI3K/AKT cascade for the ARDS category relative to controls. Enrichment appeared likewise in the PI3K/AKT cascade for the group integrating ARDS alongside CGA when juxtaposed against the ARDS category alone (Figure 6(D–E)). Convincing substantiation emerges from our comprehensive evaluation, spanning initial forecasts *via* network pharmacology to confirmatory experiments, that underscores the pivotal engagement of the PI3K/AKT cascade in the pathogenesis of ARDS provoked by LPS. Of greater significance, mitigation of pulmonary damage by CGA manifests through a mechanism tied to its capacity for restraining cascade stimulation, supported by diminished AKT alongside PI3K phosphorylation observed in pulmonary specimens. Determination *via* WB ensued for abundance of principal elements within the PI3K/AKT cascade, demonstrating markedly elevated abundance for AKT1 in conjunction with PI3K amid the ARDS category versus controls (*p* < 0.0001), whereas abundance for AKT1 together with PI3K proved substantially diminished in the cohort merging ARDS with honeysuckle’s effective constituent relative to the ARDS category (*p* < 0.0001) (Figure 6F). Indications from such observations point toward participation of the PI3K/AKT cascade in pulmonary harm linked to ARDS, alongside safeguarding of lungs afforded by honeysuckle’s chief effective element CGA, potentially constituting the primary mechanistic basis.

### Molecular docking

**Figure 7.**
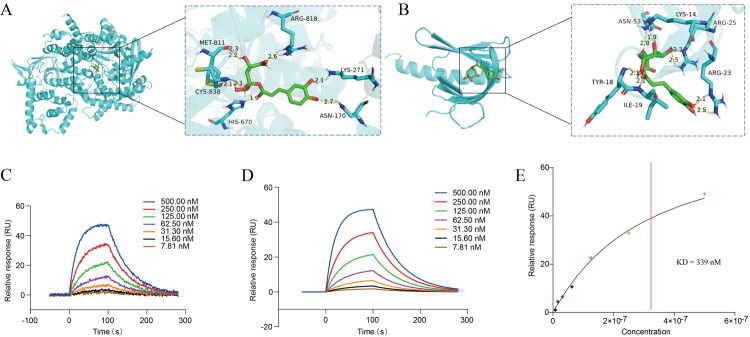
Molecular docking and SPR analysis between CGA and PI3K/AKT signaling pathway.

Molecular docking revealed a binding affinity between CGA and PI3K measuring −8.1 kcal/mol; affinities below −7 kcal/mol signify robust engagement with the protein’s active site, alongside effective accommodation of small molecules within this site through favorable conformational alignment. Visualization *via* PYMOL facilitated interaction scrutiny, disclosing hydrogen bond formations by the compound with residues ASN170, LYS271, ARG818, HIS670, CYS838, and MET811 as per three-dimensional evaluation. For AKT, the affinity registered at −6.2 kcal/mol, with values under −5 kcal/mol denoting proficient attachment to the protein’s active site, complemented by suitable integration of small molecules into this site *via* conformational compatibility. With better shape matching, followed by interaction analysis using PYMOL, and 3D analysis, it was found that the compounds formed hydrogen bonding interactions with amino acids ASN53, TYR18, ILE19, LYS14, ARG25, and ARG23 (Figure 7(A–B)). These interactions allow the compounds to bind better to the protein pocket for further action. The SPR analysis demonstrated concentration-dependent binding of Chlorogenic acid to immobilized PI3K. The kinetic parameters derived from the sensorgrams are summarized below: Association rate constant (ka): 6.74 × 10^4^M^−1^s^−1^, Dissociation rate constant (kd): 1.87 × 10^2^s^−1^, Equilibrium dissociation constant (KD): 2.77 × 10^−7^ M, Chi^2^ value: 0.98 RU^2^(Figure 7(C–E)). The low Chi^2^ value indicates a good fit of the data to the binding model. The results confirm that Chlorogenic acid binds to PI3K with high affinity, as reflected by the nanomolar-range KD value.

### Molecular dynamics simulations to analyse the compatibility and stability of chlorogenic acid binding to PI3K/AKT

**Figure 8.**
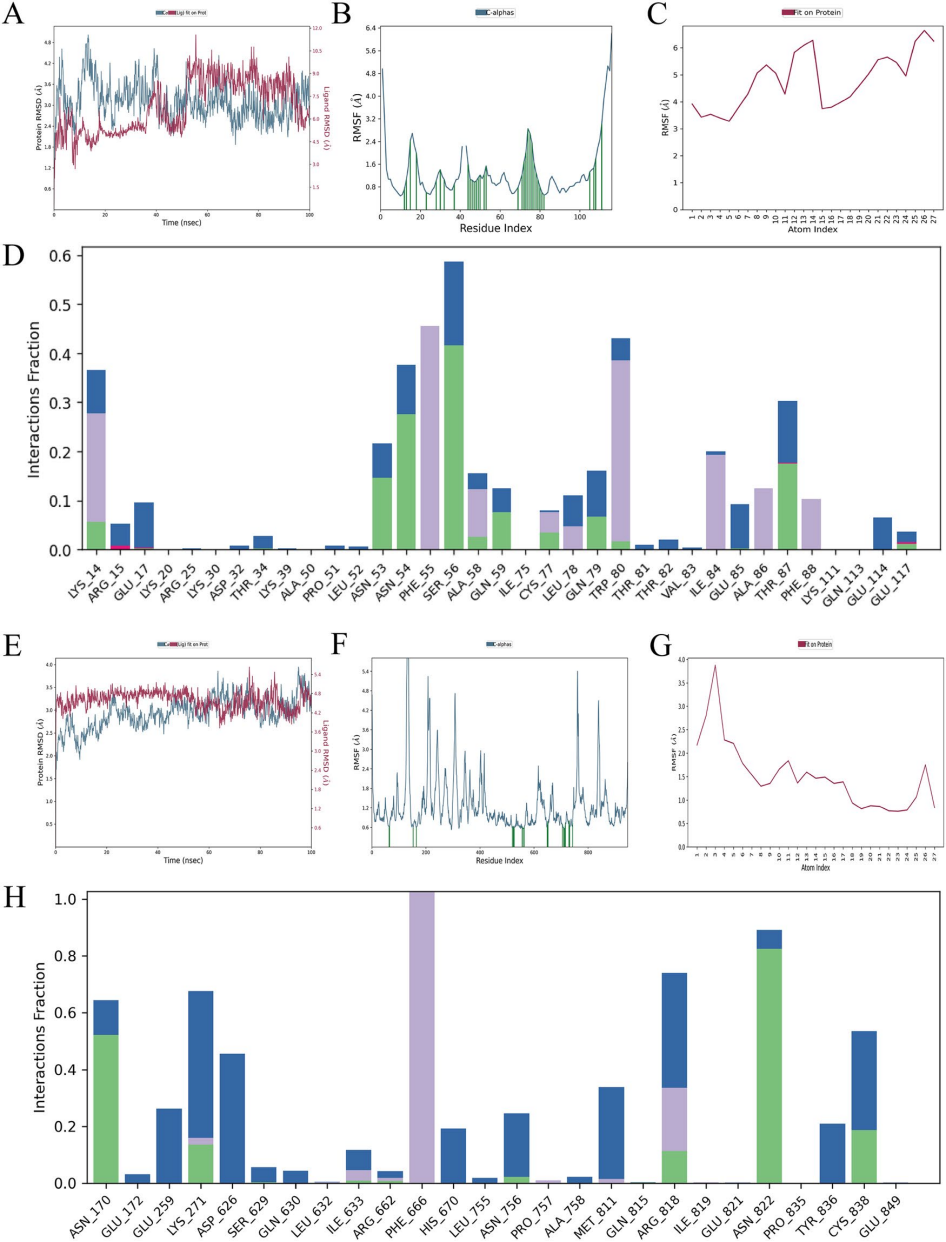
Molecular dynamics simulations to analyze the compatibility and stability of chlorogenic acid binding to PI3K/AKT. A-D: RMSD; RMSF; Ligand Root Mean Square Fluctuation (RMSF) Plot (100 ns); and Amino Acid Residue Interaction Scoring Profile for PI3K; E-H RMSD; RMSF; Ligand Root Mean Square Fluctuation (RMSF) Plot (100 ns); and Amino Acid Residue Interaction Scoring Profile for AKT.

The RMSD analysis revealed that the protein-ligand complex achieved structural equilibrium during the 100 ns simulation. The protein backbone stabilized within ∼20 ns, while the ligand attained conformational convergence after 40–50 ns, with full system equilibrium observed by 50 ns (Figure 8(A,E)). RMSF profiles further elucidated residue-specific dynamics (Figure 8(B–C, F–G)). Notably, flexible loop regions of PI3K (C-/N-termini) and AKT exhibited moderate fluctuations (RMSF ≤ 7 Å), consistent with functional domain mobility. However, ligand-binding regions remained stable, with atomic displacements ≤4 Å for PI3K (mean interatomic distance: 2.5 Å) and ≤7 Å for AKT1 (mean interatomic distance: 3 Å), indicating robust ligand adaptability within the dynamic protein environment.Interaction Analysis: CGA engaged PI3K/AKT through multiple binding modalities:

Direct Hydrogen Bonds: ASN170, ASN822, CYS838, LYS271, and ARG818 (PI3K); LYS14, ASN53, ASN54, SER56, GLN59, GLN79, and THR87 (AKT1).
Hydrophobic Interactions: PHE666, ARG818 (PI3K); LYS14, PHE55, ALA58, TRP80, ILE84, ALA86, and PHE88 (AKT1).
Water-Mediated Hydrogen Bonds: GLU172, GLU259, LYS271, ASP626, HIS670, ASN756, MET811, ARG818, TYR836 (PI3K); LYS17, LEU78, GLU85, GLU114 (AKT1). (Figure 8(D–H))

**Figure 9.**
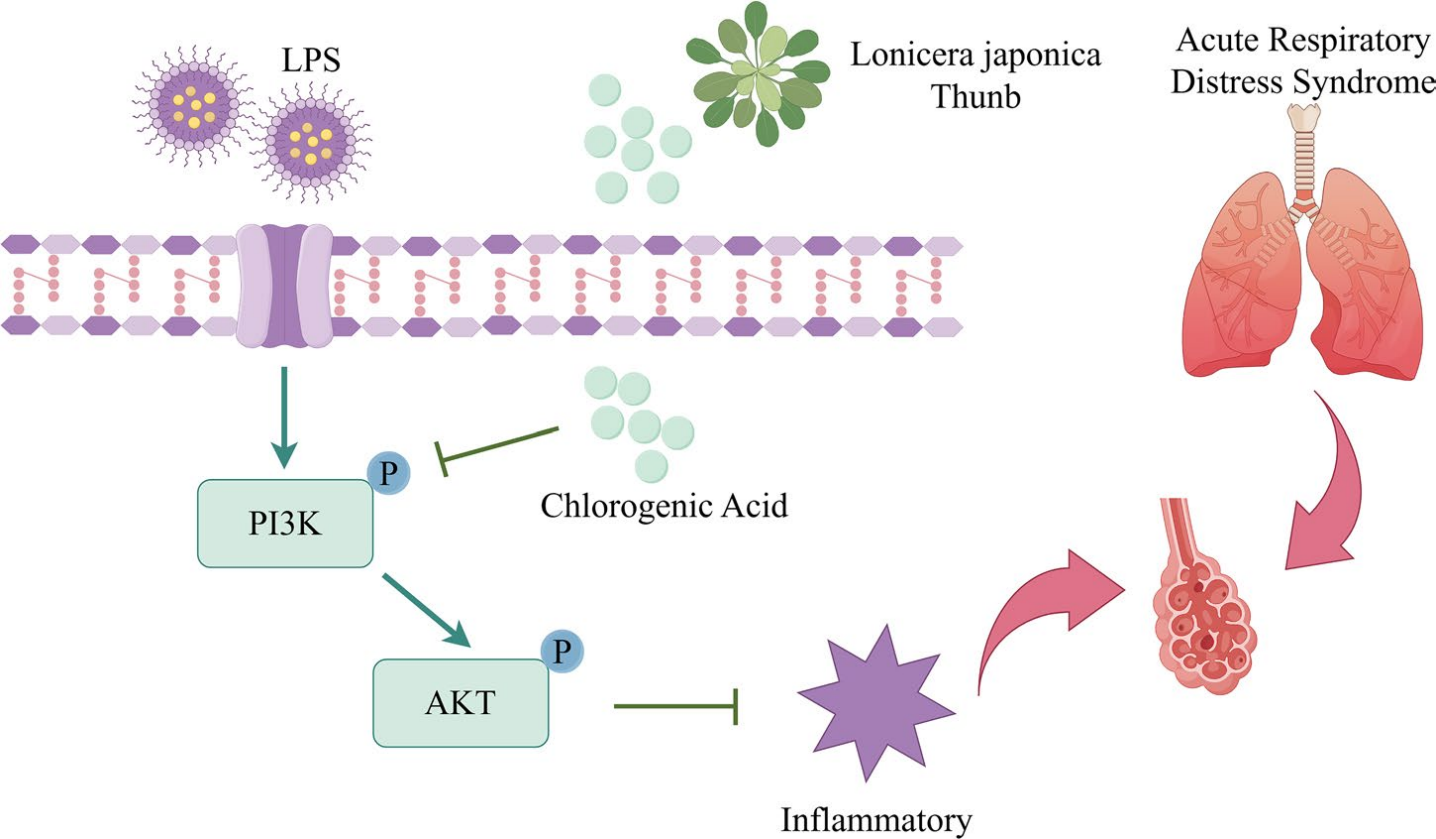
The mechanism of action of chlorogenic acid in protecting against ARDS.

These interactions collectively stabilized CGA within the PI3K/AKT binding pockets, with minimal positional drift (RMSD < 2Å post-equilibrium), underscoring its high-affinity binding and structural compatibility. And the Figure 9 presents a schematic representation of our hypothesis for CGA alleviate ARDS’ acute lung injury.

## Discussion

Among those afflicted with acute respiratory distress syndrome (ARDS) alongside hematologic malignancies, complications tied to therapy, specifically pulmonary infections, manifest frequently. Such occurrences function as a primary and deadly element that drives entries into intensive care units (ICU). Beyond managing the primary disease, addressing lung injury in ARDS is crucial for reducing the mortality rates. Current ARDS treatment strategies primarily focus on mechanical ventilation and pharmacological interventions. In recent years, clinicians have emphasized the importance of mitigating the production of proinflammatory factors in the lungs to prevent excessive inflammatory activation of lung tissues. Promising outcomes have been demonstrated through application of neutrophil elastase inhibitors for effective management of ARDS precipitated by COVID-19. Substantial involvement is exhibited by traditional Chinese medicine in addressing ARDS amid the COVID-19 outbreak, attributable to its varied effective constituents alongside extensive remedial impacts.

Preliminary network pharmacology analysis revealed a common target between ARDS and honeysuckle, prompting the investigation to investigate the primary active ingredient of honeysuckle, as a potential modulator of lung injury. By constructing an LPS-induced ARDS model and assessing T-cell subpopulations, we aimed to verify the molecular signalling pathways involved in high-throughput sequencing and western blot (WB) analysis. Evidence from our investigation reveals 144 overlapping objectives between ARDS and the principal effective constituent derived from honeysuckle, encompassing leading 20 essential genes such as SRC, AKT1, PI3KCA, and STAT3. Despite limited investigations emphasizing application of honeysuckle alongside its primary elements for managing COVID-19[17,23], inhibition of cytokine storms linked to novel coronavirus pneumonia in conjunction with SARS-CoV-2 penetration was illustrated by honeysuckle in an *in vitro* setting, as reported by Yeh et al. [20]. Elucidation is still required for precise mechanistic underpinnings, which implies potential attenuation by honey suckle of inflammatory imbalance alongside COVID-19 contagion hazards.

Involvement of molecular signalling cascades such as HIF, Ras, PI3K/AKT1, and others within overlapping objectives shared between ARDS and principal effective elements from honeysuckle was disclosed through examination *via* functional enrichment. Reduction in apoptosis alongside autophagy amid ARDS precipitated by sepsis is achieved *via* ginseng injection through suppression of the PI3K-AKT cascade, as forecasted by Chen et al. [13]. Mitigation of acute pulmonary damage triggered by bleomycin has been exhibited by thorny defense granules *via* the PI3K/Akt/mTOR cascade [23]. Attenuation of pulmonary harm induced by LPS occurs through melatonin *via* restraint of HIF-1α/GLUT1/NLRP3, as illustrated by Zheng et al. [23]. Pivotal contributions are rendered by these cascades implicated in shared objectives between ARDS and honeysuckle toward both progression alongside pathogenesis of ARDS and prospective remedial effectiveness exhibited by honeysuckle’s chief elements, as implied by such observations.

An LPS-induced rat model was established, followed by delivery of honeysuckle’s principal effective elements, to ascertain safeguarding influences exerted by these constituents upon pulmonary damage triggered by LPS in ARDS. Observations demonstrated substantial elevations within the ARDS cohort relative to controls regarding oedema magnitude, cellular infiltration, and interstitial thickness. Nevertheless, notable diminutions in those metrics materialized subsequent to CGA administration amid an ARDS scenario provoked by LPS. Substantial diminutions ensued for CD8+ alongside CD4+ T cells after CGA administration, contrasting with their pronounced augmentation observed in the LPS-triggered ARDS scenario versus controls. In opposition, marked augmentations emerged for CD25 + Foxp3+ T cells across ARDS model cohorts as well as controls post CGA administration. Determinations through ELISA targeting mediators of inflammation within bronchoalveolar lavage fluid uncovered augmented concentrations for IL-10, IL-6, IL-1β, and TNF-α amid the ARDS cohort compared against controls, yet substantial reductions in such concentrations occurred within the ARDS cohort subjected to CGA. Greater disruption to subcellular architecture in pulmonary specimens became apparent *via* examination with transmission electron microscopy for the ARDS cohort as opposed to cohorts treated with CGA in ARDS alongside controls.

Contrary to our findings, Adamzik et al. reported a positive correlation between elevated proportions of CD25 + Foxp3+ T cells in the alveoli and 30-day mortality in ARDS patient [24]. However, Singer BD proposed a different perspective, suggesting that CD25 + Foxp3+ Tregs could protect against lung injury and promote lung injury repair, as observed in previous studies [25–27]. The role of CD25 + Foxp3+ T cells in lung injury requires further confirmation through additional research. Wang et al. systematically elucidated the role of CD4+ T cells in the production and resolution of inflammation in ARDS[24]. Crucial involvement has been demonstrated for CD4+ T cells amid pulmonary damage progression, wherein substantial contributions are made by inflammation resolution alongside its emergence, yet elucidation remains necessary for their exact functions [28]. Promotion of inflammatory reactions by T cells during pneumonia caused by severe acute respiratory syndrome coronavirus 2, coupled with establishment of positive feedback circuits involving the virus, received confirmation from Grant et al. [29]. Modulation of immune cell subset categorization by CGA is implied through our observations, consequently affording defense against pulmonary harm in ARDS, given that inflammatory mediators originate from effector immune cells. Comprehensive exploration has yet to address the function of T cell subsets within ARDS, necessitating immediate additional inquiries to substantiate linkages between lung inflammation in ARDS and T cell subsets. Nevertheless, pro-inflammatory contributions potentially arise from CD8+ alongside CD4+ T cells according to hypotheses in our investigation, whereas excessive activation within the immune system undergoes suppression *via* CD25 + Foxp3+ T cells, resulting in inflammation that fosters cytokine storms subsequent to CGA administration. Inference of this derives from elevated concentrations of pro-inflammatory mediators in conjunction with CD8+ and CD4+ within serum alongside alveolar lavage fluid amid ARDS, in addition to downregulation of related pro-inflammatory mediators together with CD8+ and CD4+ post CGA administration, culminating in pulmonary damage.

In addition to immune cell activation leading to inflammatory cytokine storms and subsequent lung injury, pathogenic infections or tumors can also trigger the formation of neutrophil extracellular trap (NET) networks. These networks can further impair lung microcirculation, forming immune thrombi that prevent pathogen spread, while also producing microthrombi, exacerbating lung injury. Consequently, current clinical studies on patients with ARDS are exploring the use of neutrophil elastase inhibitors to inhibit NET formation and mitigate lung injury. Higher concentrations of citH3 and PAD4 were detected in the ARDS cohort compared to controls through ELISA assays on serum and alveolar lavage fluid samples, showing a decrease following CGA treatment. As primary defenders against infections and essential elements of innate immunity, neutrophils eliminate pathogens by employing processes like degranulation, phagocytosis, release of antimicrobial peptides, and generation of reactive oxygen species. Nevertheless, overabundant NET production may harm endothelial and epithelial cells in the lungs through elements such as DNA, cathepsin G, histones, NE (neutrophil elastase), and MPO (myeloperoxidase). Research indicates a positive association between plasma NETs in individuals with ARDS and both mortality and disease intensity, functioning as an indicator for prognosis [30–32]. Thus, suppressing surplus NET generation represents a promising strategy for treating ARDS [33–35]. In a rat model of ARDS, CGA—a principal constituent of honeysuckle—has been shown to reduce NET formation.

We further investigated the potential molecular mechanisms underlying ARDS injury and the protective effects of CGA, the main component of honeysuckle, on ARDS lung injury. GO/KEGG enrichment analysis of mRNA high-throughput sequencing of lung tissues revealed the presence of the PI3K/AKT molecular signalling pathway in ARDS compared to the control group and its modulation by CGA intervention. Combining this with the initial network pharmacological analysis of the signalling pathway shared by the honeysuckle main ingredient and ARDS, we posit that PI3K/AKT is a key component in ARDS pathogenesis. PI3K/AKT is a principal molecular signaling pathway involved in the development of and protection against ARDS lung injury with honeysuckle’s main ingredient. We measured the expression levels of the key factors in this pathway to further elucidate their role. The results indicated that PI3K/AKT was upregulated in the ARDS rat model, but downregulated after CGA intervention. The inhibitory effect of CGA on the PI3K/AKT signalling pathway has been well-documented across diverse pathological contexts, underscoring its broad therapeutic potential. For instance, in cancer biology, Alzahrani et al. demonstrated that CGA suppresses the proliferation and metastasis of MDA-MB-435s breast cancer cells by downregulating the PI3K/AKT/mTOR axis, highlighting its role in attenuating oncogenic signalling cascades[36]. Similarly, in hepatic pathology, CGA ameliorates carbon tetrachloride (CCl4)-induced liver fibrosis through selective inhibition of PI3K/AKT activation, thereby reducing extracellular matrix deposition and fibrogenic gene expression [37]. Beyond these findings, CGA exerts neuroprotective effects in cerebral ischemia-reperfusion injury by modulating PI3K/AKT signalling to attenuate neuronal apoptosis and oxidative stress [38]. Furthermore, in gastrointestinal disorders, CGA mitigates intestinal epithelial injury under oxidative stress by enhancing AKT-mediated survival pathways, which stabilize mucosal barrier integrity [39]. These collective observations across disparate disease models corroborate the conserved role of PI3K/AKT signalling as a pivotal target of CGA, reinforcing its mechanistic relevance in ARDS pathophysiology. He et al. demonstrated that CGA protects against ARDS lung injury by enhancing phagocytosis of alveolar macrophages through the activation of G-protein-coupled receptor 37 (GPR37) and inhibiting excessive inflammation [40]. Jain et al. revealed that CGA inhibits TLR4/3, attenuates oxidative stress-mediated NLRP3/NF-κB axis, and ameliorates LPS + POLY I: C-induced ARDS [41]. Li et al. showed that MicroRNA-877-5p enhances the PI3K/Akt pathway by targeting CDKN1B, alleviating ARDS *in vivo* and *in vitro* [42], suggesting that PI3K/AKT activation may play a protective role against ARDS. However, Sun et al. concluded that Jing Fang Granules attenuate bleomycin-induced acute lung injury by downregulating the PI3K/Akt/mTOR signalling pathway [23]. Another study revealed that Shen Fu injection inhibited inflammation and oxidative stress by regulating autophagy and apoptosis in sepsis-induced ARDS by inhibiting the PI3K-AKT pathway, thereby protecting against lung injury [43]. These contradictory results require further experimental validation. PI3K/AKT is associated with immune system regulation and inflammation production and predominantly plays a pro-inflammatory role [44–46]. Additionally, the PI3K/AKT signalling pathway is a primary regulator of NET formation; the total saponin of Dioscorea perforata inhibits neutrophil extracellular trap formation through the PI3K/AKT/mTOR axis, alleviating gouty arthritis in rats [47]. Given the importance of the PI3K/AKT signalling pathway in ARDS pathogenesis and development, studies have been conducted to elucidate its potential role and therapeutic potential in severe acute respiratory syndrome coronavirus type 2-induced coagulopathy [48]. We performed molecular docking between CGA, a major component of honeysuckle, and PI3K/AKT1 to confirm that CGA can effectively bind to PI3K/AKT1 and exert biological effects. The results confirmed that CGA has a strong binding affinity for PI3K/AKT1, which underpins its biological activity through this pathway. **This study has several limitations. Firstly, although the evidence supporting CGA’s direct inhibition of the PI3K/AKT pathway has received support from multiple aspects, it lacks** *in vitro* **cell validation. The reduction of PI3K/AKT phosphorylation observed** *in vivo* **may be secondary to the overall attenuation of inflammation. Further research on the relevant cell lines is a necessary condition for determining direct molecular targeting. Secondly, the treatment plan for CGA (7 days before and after treatment) is more preventive than therapeutic, and may not fully reflect the clinical situation where treatment is initiated only after the onset of the disease. The clinical translatability of our research results will be enhanced through future experiments using post-onset intervention models.** Finally, the specific role of the PI3K/AKT molecular signalling pathway in ARDS needs to be further verified through rescue experiments.

Based on this evidence and the results of this study, we conclude that CGA, the main component of honeysuckle, protects against ARDS lung injury by inhibiting PI3K/AKT1, thereby suppressing excessive immune cell activation, inflammation dysregulation, and NET formation in ARDS. Given the low cost and multiple mechanisms of action of honeysuckle and its main components, these findings may offer potential clinical value for the treatment of ARDS. However, attention must also be paid to possible adverse effects.

## CRediT authorship contribution statement

Jie Wei and Guan Ye Nai: Writing – original draft and project administration. Min Wu: Formal analysis and data curation. Yu Mei Huang, Zhao Ping Gan, and Zhen Bin Wei: Software, Methodology. Wei Jie Zhou: Writing-review and editing, validation. Hui Li and Rong rong Liu: Writing – review and editing, supervision. All authors have read and approved the final version of the manuscript.

## Data Availability

All data underlying this study are available with request made to the corresponding author of the manuscript.
